# The Voter’s Guide

**Published:** 1896-10

**Authors:** 


The Voter's Guide. A digest of the election laws of Pennsylvania, compiled by Hon. Jesse M. Baker, author of the Baker Ballot Law. Published by Wm. G. Johnston & Co., Penn Avenue and Eighth Street, Pittsburg, Pa. 1896. Price, in paper cover, 25 cents.

This book is interesting only to Pennsylvanians and those outside of Pennsylvania who contemplate a reform in the election laws of their own States.

Pennsylvania has made a complete revolution in her method of voting by adopting the famous "Australian System." This book gives a minute explanation of that system, aided by diagrams, wood-cuts, and *fac similes* of the ticket. Every voter should have a copy of this book, and thus make himself familiar with all the machinery of the precious American right of suffrage.

